# Comparative evaluation of ramie, hemp, and hollow fibers on the mechanical properties of MICP-treated calcareous sand

**DOI:** 10.3389/fmicb.2026.1869298

**Published:** 2026-07-20

**Authors:** Maleck Massou, Noel Babu, Jun Hu, Zhang Shuai, Hanyu Dang, Wu Yuwei

**Affiliations:** 1 School of Civil Engineering and Architecture, Hainan University, Haikou, China; 2 School of Civil Engineering, Harbin Institute of Technology, Harbin, China; 3 School of Information and Communication Engineering, Hainan University, Haikou, China; 4 Department of Infrastructure Engineering, The University of Melbourne, Parkville, VIC, Australia

**Keywords:** calcareous sand, hollow fiber membrane, mechanical properties, MICP, natural fiber

## Abstract

A combination of fiber reinforcement and microbially induced carbonate precipitation (MICP) has emerged as a highly promising, sustainable method for improving the mechanical properties of calcareous sands. This study investigates the effect of three fiber types, hemp (HF), ramie (RF), and hollow fiber (HoF), on the mechanical properties of MICP-treated calcareous sand. Cylindrical samples with fiber content of 0.2, 0.3, 0.4, 0.5, and 0.6% by weight of sand were prepared and tested for water absorption, bacterial retention, unconfined compressive strength (UCS), and calcium carbonate content. The results indicate that MICP effectively cemented the calcareous sand, and the inclusion of fibers further enhanced its strength and ductility, while mitigating brittle failure. The bacterial retention rate of hollow fiber was the highest at 30.5% (114.8% increase over the control), followed by ramie fiber at 26.0% (83.1% increase), and hemp fiber at 21.0% (47.9% increase). This trend was attributed to the microporous structure of the hollow fiber, which provides abundant attachment sites for bacterial adhesion. The optimum UCS was achieved at 0.3% fiber content for hemp and ramie fibers and 0.5% for hollow fibers. At these contents, the UCS increased by 12.82, 46.83, and 59.54%, respectively, compared to the control. The change in UCS closely followed the trend of calcium carbonate precipitation, with the hollow fiber reinforced samples showing the highest CaCO₃ content due to improved bacterial retention and nucleation. Scanning electron microscopy (SEM) observations revealed different interfacial bonding mechanisms between the materials. The hollow and ramie fibers formed dense calcite coatings, which bonded strongly to the sand matrix, whereas the hemp fibers showed limited calcite formation, resulting in poor interfacial strength.

## 1 Introduction

Calcareous sand, characterized by irregular morphology, high internal porosity, and low compressive load-bearing capacity, constitutes the predominant foundation soil throughout the South China Sea (Zhao et al., 2021). As a result, the soil foundation is highly susceptible to failure under cyclic loading, necessitating the adoption of new approaches to enhance its geotechnical properties. Chemical and mechanical techniques are the primary methods for soil stabilization, with cement being the most conventional and widely used material in chemical applications (Yazici and Keskin, 2024). However, common foundation-reinforcement methods, such as layered rolling, pile foundation treatment, or replacement, are hindered by significant defects, high energy consumption, high costs, and substantial environmental impacts (Anagnostopoulos et al., 2011; Al Qabany and Soga, 2014). In response to these challenges, various biological processes, such as biogas production, biofilm formation, and bio-cementation, have been recommended to overcome certain drawbacks of conventional soil improvement techniques (Dejong et al., 2014). These techniques no longer view soil as an inert material but rather as a living ecosystem. Among these, Microbial-Induced Calcite Precipitation (MICP) has emerged as a promising technique for ground improvement. A large number of studies have shown that MICP can significantly improve the stiffness, shear strength, and compressive strength of weak soils (DeJong et al., 2010; Feng and Montoya, 2016). The MICP technique uses ureolytic bacteria, specifically *Sporosarcina pasteurii*. This strain hydrolyzes urea (NH_2_)_2_CO into ammonium (NH₄^+^) and carbonate (CO_3_^−2^) ions. The latter can then contribute to the formation of the final product, calcite (CaCO_3_), in a calcium-rich (Ca^2+^) environment as seen in Equation (1).

$${\left({\mathrm{NH}}_{2}\right)}_{2}\mathrm{CO}+2H_{2}O+{\text{CaCl}}_{2}\to {\text{CaCO}}_{3}+2{\mathrm{NH}}_{4}\mathrm{CL}$$ (1)

Unlike chemical grouts, biocementation is not limited by a setting time or by an increase in fluid viscosity during injection. As a result, it is a competitive alternative to conventional techniques, especially when addressing large-scale areas that require a quick solution. Furthermore, biocementation is considered more environmentally friendly, as it can reduce CO_2_ emissions, thereby contributing to mitigating global warming (DeJong et al., 2010; Xiao et al., 2018). Additionally, the energy required to produce calcite by bacteria accounts for 10% of the total energy needed to produce the same quantity of conventional cement (Feng et al., 2015). Over the last decade, the effectiveness of MICP has been demonstrated through numerous laboratory tests (Al Qabany et al., 2012; Montoya et al., 2014) and large-scale field applications (Filet et al., 2012). Recent studies have explored MICP applications in calcareous sand under various environmental conditions, including the domestication of bacteria in seawater (Wang et al., 2024). However, the major obstacle to the widespread adoption of MICP soil treatment is its quasi-brittle failure mode, attributed to the irregularity of carbonate precipitation (van Paassen et al., 2010; DeJong et al., 2006). To address this problem, researchers have proposed integrating fibers into the MICP soil reinforcement process (Jiang and Soga, 2016; Liu et al., 2017). Fiber reinforcement is a traditional, eco-friendly technique for soil stabilization that effectively improves mechanical properties, notably toughness and ductility (Maher and Gray, 1990; Consoli et al., 2003). However, fiber type, aspect ratio, and interfacial bonding all strongly affect the mechanical response (Feng and Montoya, 2016). The reinforcement effect is influenced by the effective bonding between the fiber and the material, specifically the interfacial shear resistance (Dove and Frost, 1999; Tang et al., 2010). Several studies have combined the MICP technique with fiber reinforcement to enhance the mechanical strength of quartz sand, particularly the unconfined compressive strength and splitting tensile strength (Al Imran et al., 2020; Zhao et al., 2020). Tang et al. and Dubey et al. reported that the integration of randomly dispersed fibers can significantly enhance the shear strength and increase the soil ductility (Tang et al., 2022; Dubey et al., 2022). For instance, Zeng et al. (2021) investigated the unconfined compressive strength (UCS) of fiber-reinforced silica sand by varying the cement-to-sand ratio and the polyvinyl alcohol (PVA) fiber ratio. The results showed that samples with a 2% cement ratio had the greatest increase in UCS, more than tripling at a fiber ratio of 1%. Zeng et al. (2021) evaluated the tensile strength of biocemented calcareous sand with varying polypropylene fiber contents, concluding that excessive fiber content has a detrimental effect on tensile strength. The studies mentioned earlier have primarily focused on the effects of adding a single type of fiber to biocemented soils. However, an overall understanding of the impact of different categories of fibers, such as natural, synthetic, or inorganic, on the properties of MICP-treated sand remains insufficient. Furthermore, while significant research has explored the unconfined compressive performance strength of fiber-reinforced biocemented sand, a key factor in limiting brittle failure remains poorly studied. To address these gaps, this study compares three fiber types (hemp, ramie, and hollow) at different fiber contents (0.2–0.6% by weight of sand) to determine the optimum fiber content for each fiber type, evaluate mechanical characteristics (UCS, CaCO₃, water absorption, bacterial retention), and identify the most promising fiber for calcareous sand improvement by understanding how fiber type influences bacterial capture and MICP efficiency. The research results are expected to provide technical support and a reference for MICP applications in South China Sea island and reef reinforcement projects.

## 2 Materials and methods

### 2.1 Materials

#### 2.1.1 Calcareous sand

**Figure 1:**
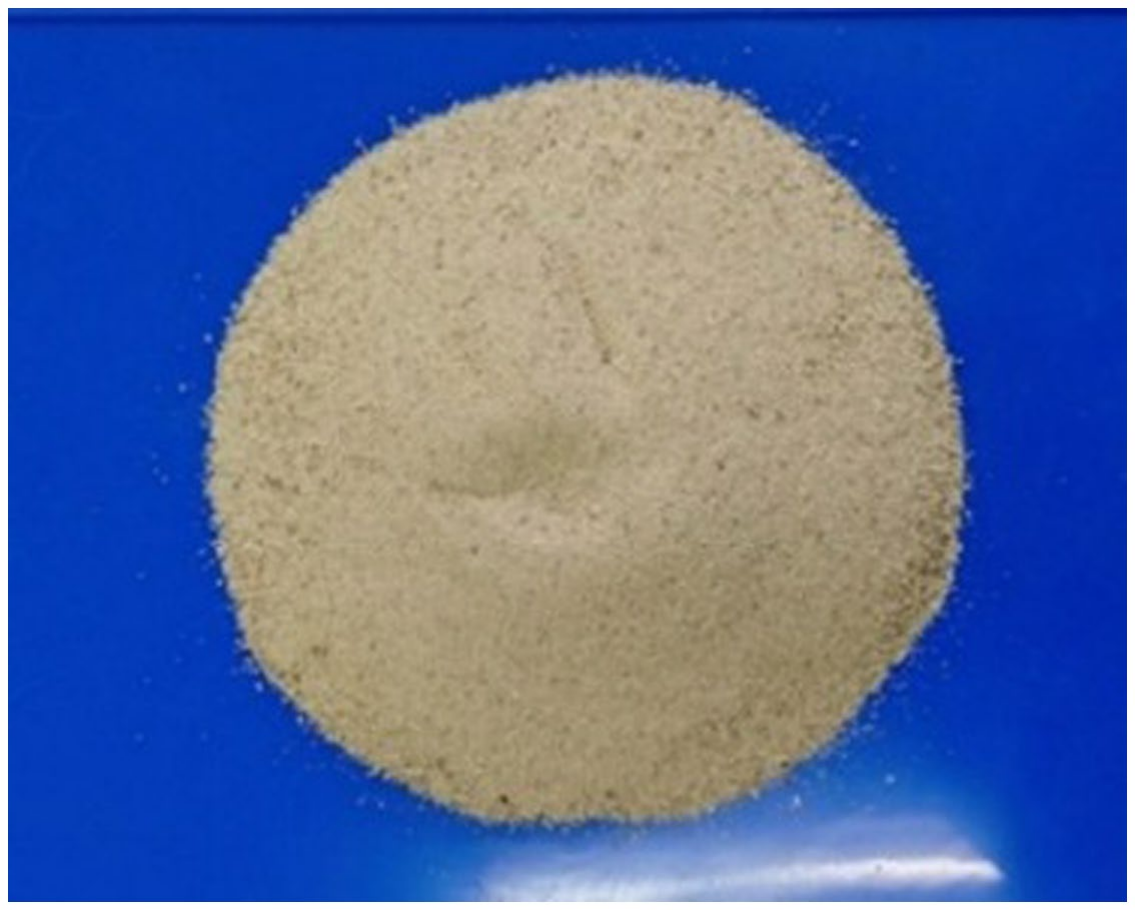
Calcareous sand used in this study.

The sand used in this study was collected from an island in Sansha City, Hainan Province Figure 1. The raw sand sample was washed, dried, cooled, and purified of impurities to obtain the test sand. The parameters of the calcareous sand are presented in Tables 1, 2. All parameter measurements were conducted in accordance with the Standard for Geotechnical Testing Methods (GB/T 50123–2019). The calcium carbonate content of the test sand is 62.9% (exceeding 50%), classifying it as calcareous.

**Table 1:** The basic physical properties of the calcareous sand.

Properties	Value
Non-uniformity coefficient	4.484
Coefficient of curvature (C_c_)	0.76
Specific gravity (G_s_)	2.618
Maximum dry density (g/cm^3^)	1.702
Minimum dry density (g/cm^3^)	1.379
Maximum void ratio	0.91
Minimum void ratio	0.54

**Table 2:** Test sand specific composition.

Mineral composition	CaCO_3_	SiO_2_	Ca_2_(SO_4_)_2_(H_2_O)	Others
Mineral content	62.9%	24.5%	7.6%	5%

#### 2.1.2 Fiber types

**Figure 2:**
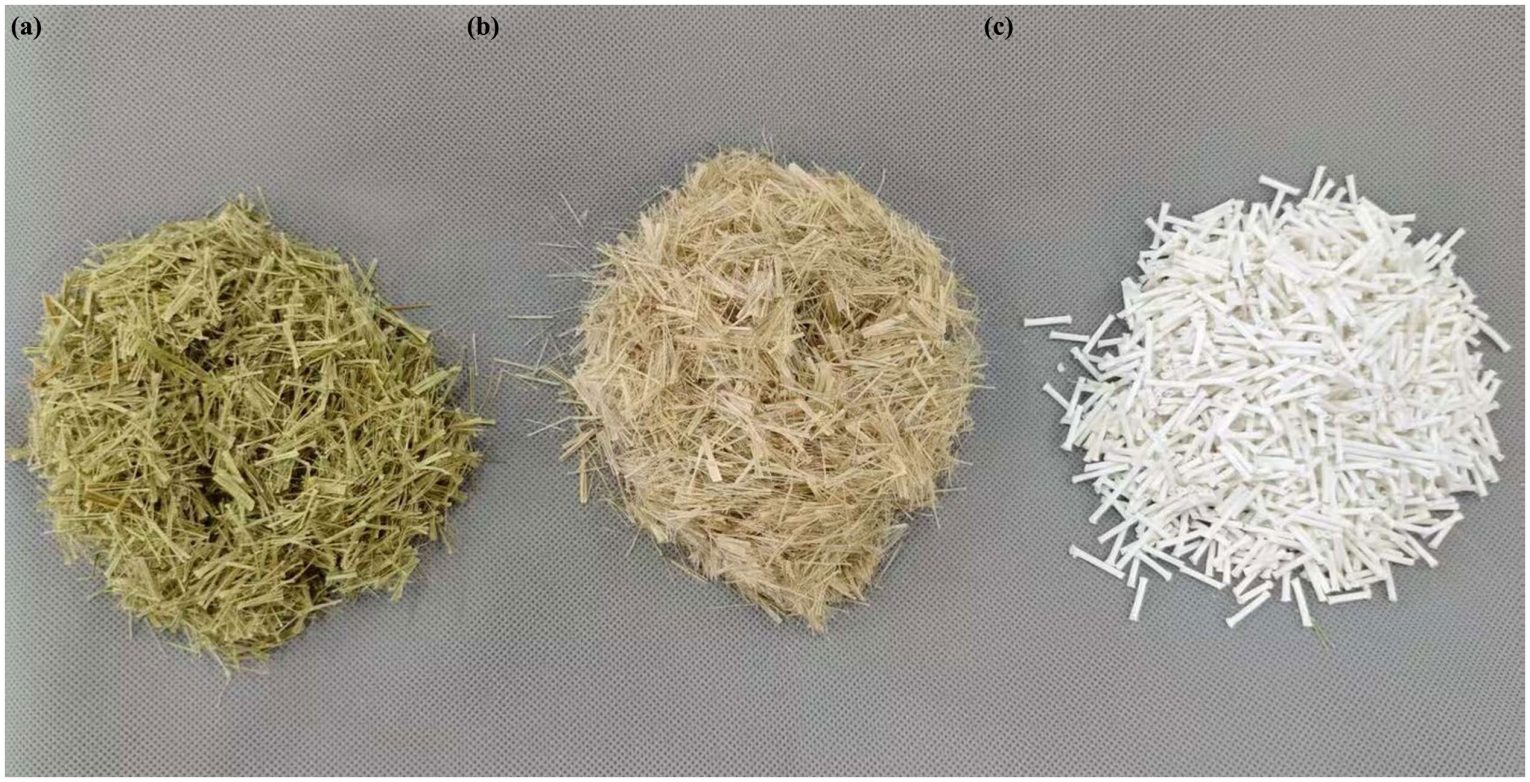
Cut fibers. **(a)** Ramie fiber, **(b)** hemp fiber, **(c)** hollow fiber.

In this research, Hemp fiber (HF), Ramie fiber (RF), and Hollow fiber (HoF) were selected. Hemp fiber belongs to the natural plant fiber category, which is more widely distributed and easier to obtain than animal fiber. The mechanical properties of these fibers are listed in Table 3, and their photos are shown in Figure 2.

**Table 3:** Physical and chemical properties of the fibers.

Parameters	Ramie fiber	Hemp fiber	Hollow fiber
Color	Green	Yellow	White
Length (mm)	10	10	10
Tensile Strength (MPa)	400	650	2,350
Modulus of elasticity (GPa)	80	70	60
Elongation at break (%)	1.7	1.6	–
Density (g/cm^3^)	1.5	1.4	–

Values not provided by the manufacturer.

### 2.2 Specimen preparation

#### 2.2.1 Bacteria and cementation solution

The strain used in this study was *Sporosarcina pasteurii* obtained from the Guangdong Microbial Culture Collection Center (GDMC). It is a non-pathogenic, Gram-positive bacterium commonly found in soil (DeJong et al., 2006). As a high-yield urease strain, *Sporosarcina pasteurii* can produce a large amount of urease during its metabolism to promote urea hydrolysis, and the reaction process is environmentally friendly. Based on previous research (Xu et al., 2021; Chu et al., 2012), the strain was cultured aerobically in a sterile liquid medium (pH 9.0) containing, per liter: 10 g ammonium sulfate, 20 g yeast extract, 0.024 g nickel chloride, and 0.01 g manganese sulfate. Bacterial cultures were grown at 28 °C for 30 h in a conical flask on an orbital shaker (200 rpm). The urease activity was measured for bacterial samples at an optical density of 1.7–2 using a conductivity method, and the activity was determined to be approximately 4.8 mM urea/min. In this experiment, the cementation solution was prepared by dissolving calcium chloride (CaCl₂) and urea (CH₄N₂O) in deionized water, each at 0.5 mol/L.

#### 2.2.2 Sample preparation

**Figure 3:**
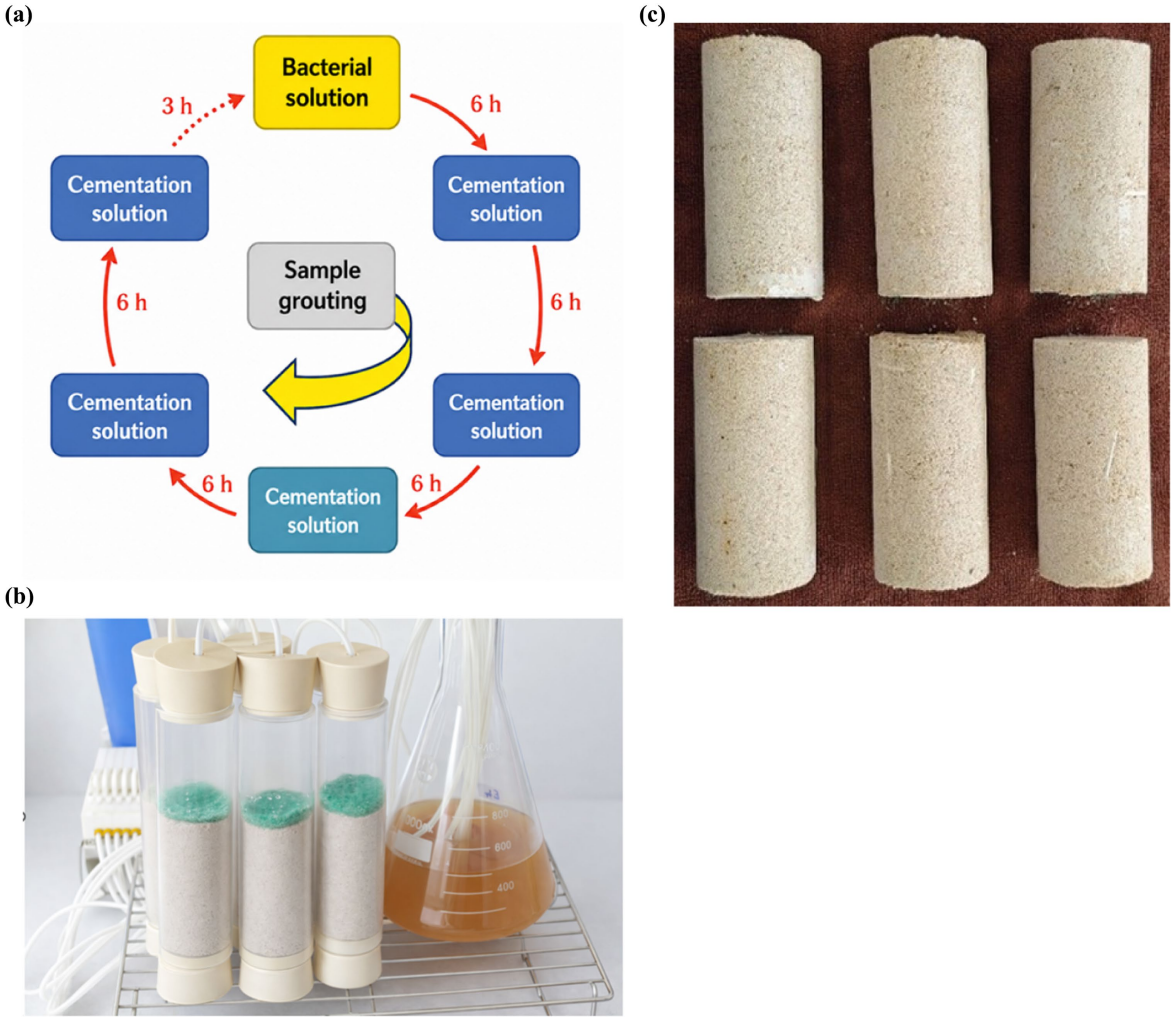
Sample preparation. **(a)** Grouting process, **(b)** sample grouting, **(c)** reinforced samples.

The mold selected for the experiment is a cylindrical acrylic mold with a height of 130 mm, an inner diameter of 39.1 mm, and a wall thickness of 3 mm. The bottom of the mold is sealed with a perforated rubber stopper. The perforations in the rubber stopper allow excess bacterial and cementation solutions to flow out during permeation after adsorption and reaction within the sample’s pores. To facilitate demolding, a pre-cut PVC film was coated with Vaseline on both sides and placed inside the mold. After weighing 128 g of calcareous sand using a balance, the designated fiber (at 0.2, 0.3, 0.4, 0.5, and 0.6% by weight of sand) was added, followed by 8 g of deionized water (approximately 5% of the calcareous sand mass). The fiber content range of 0.2 to 0.6% by weight of sand was selected based on previous research showing that contents below 0.2% produced negligible strength improvement (less than 5% increase in UCS), while contents above 0.6% caused visible fiber agglomeration and non-uniform distribution, leading to reduced UCS (Shan et al., 2022; Kou et al., 2023). This range is also similar to the ones reported in previous studies on fiber-reinforced MICP-treated sands, which typically report optimal fiber contents between 0.2 and 0.4%. After uniform mixing, the specimens were divided into three layers in the mold. Each layer was slightly compacted with a compaction tool. Before compacting each layer with a compactor, a brush was used to lightly roughen the interface between layers to prevent distinct stratification in the specimens. After sample preparation, the MICP injection process was initiated at 25 °C. The process followed these steps: (1) Inject distilled water into the sample by the peristaltic pump, to flush out the impurities in the test sand; (2) inject about a sample pore volume of bacterial solution (i.e., 40 mL); (3) after 3 h for resting, repeat the injection of 40 mL of cementing solution four times with the time interval of 6 h, and turn over the sample every two times. The grouting procedure and device are illustrated in Figures 3a,b. Photographs of the prepared reinforced samples are shown in Figure 3c.

### 2.3 Testing method

#### 2.3.1 Water absorption test

The water absorption of the biocemented sand was evaluated following the methodology of Manzur et al. (2017). This test measures a material’s capacity to absorb moisture under standard atmospheric pressure, serving as an indicator of its surface porosity. The procedure was conducted as follows: The mass of the cured and dried sample is recorded as *m_1_*. After drying, the sample is placed flat in pure water and soaked for 24 h to achieve complete saturation. The surface moisture is then wiped off with a thoroughly wrung damp towel, and the sample is quickly weighed and recorded as *m_2_*. The water absorption rate can be expressed by Equation (2).

$$w=\frac{m_{2}-m_{1}}{m_{1}}\times 100\%$$ (2)

Where *w* is the water absorption rate, *m_2_* is the mass after water absorption, and *m_1_* is the mass before water absorption.

#### 2.3.2 Calculation of calcium carbonate

The acid-washing method is often used to quantify the CaCO₃ generated in samples during the MICP process. However, the sand used in this research is calcareous sand with a high native CaCO₃ content (Feng and Montoya, 2016; Montoya et al., 2014). Acid washing with hydrochloric acid would damage the calcareous sand’s structure. Furthermore, the calcium carbonate content in calcareous sands cannot be reliably quantified by titration, X-ray diffraction, or thermogravimetric analysis due to interference from native carbonate minerals (Al Qabany et al., 2012; Choi et al., 2017). Therefore, the weighing method was adopted to measure the CaCO₃ content produced by the MICP treatment. Following the MICP reinforcement process, the sample is submerged in deionized water for 24 h to ensure that any soluble salts are fully dissolved and removed. After this soaking phase, the sample is placed in a constant-temperature oven at 55 °C for 48 h to dry completely. The final mass of the sample is measured immediately after it is removed from the oven. The content of precipitated CaCO₃ was determined using Equation (3):

$$c=\frac{m_{2}-m_{1}}{m_{1}}\times 100\%$$ (3)

Where *c* is the mass percentage of generated CaCO_3_ in treated samples, *m_1_* is the initial dry mass of the specimen before MICP treatment, and *m_2_* is the final dry mass of the specimen after MICP treatment and washing.

#### 2.3.3 Bacterial retention rate measurement

The bacterial retention rate was measured to evaluate the bacterial capture capacity of each fiber type. To avoid interference from the cementation solution, the test was conducted only during the first bacterial injection. The OD₆₀₀ value of the bacterial solution was measured before injection using a spectrophotometer and recorded as OD1. After injecting 40 mL of bacterial solution into the sand column, the effluent was collected immediately upon exiting the bottom, and its OD₆₀₀ was measured and recorded as OD_2_. The bacterial retention rate was calculated using Equation (4):

$$\mathrm{OD}=\frac{OD_{1}-OD_{2}}{OD_{1}}\times 100\%$$ (4)

Where OD is the percentage of bacteria retained in the sand column, OD₁ is the optical density of the bacterial solution before injection, and OD₂ is the optical density of the effluent collected after passing through the sand column. Three replicate measurements were performed for each fiber type and content.

#### 2.3.4 Unconfined compressive strength

The unconfined compression test is a simple and rapid method for measuring the strength of soil samples. Unconfined compressive strength (UCS) is widely used for the rapid comparison of MICP-treated samples fabricated using different protocols. The test was conducted in accordance with the American Society for Testing and Materials (ASTM) standard D2166 / D2166M for unconfined compressive strength. The prepared samples had dimensions of 90 mm in height and 39.1 mm in diameter. Testing was performed using an electronic universal testing machine (model DHT-800, Hengyi Precision Instrument Co. Ltd., Shanghai) with a controlled loading speed of 1 mm/min. Three samples were tested for each condition, and their average values were recorded.

#### 2.3.5 Microstructural analysis

To analyze the morphology of the biocemented calcareous sand at the microscopic level, scanning electron microscopy was performed on selected samples. Microstructural observations of the reinforced specimens were conducted using a Verios 5 XHR SEM, an ultra-high-resolution instrument produced by Thermo Fisher Scientific. The working principle of the SEM involves an electron beam emitted from an electron gun, which is focused onto the sample via magnetic lenses under an accelerating voltage. Various signals emitted from the sample are detected, and a computer processes their intensities to generate grayscale images. SEM was used to characterize the morphology of calcium carbonate crystals, the cementation at fiber-sand particle interfaces, and the distribution of fibers within the specimens.

## 3 Results and discussion

### 3.1 Water absorption test results

**Figure 4:**
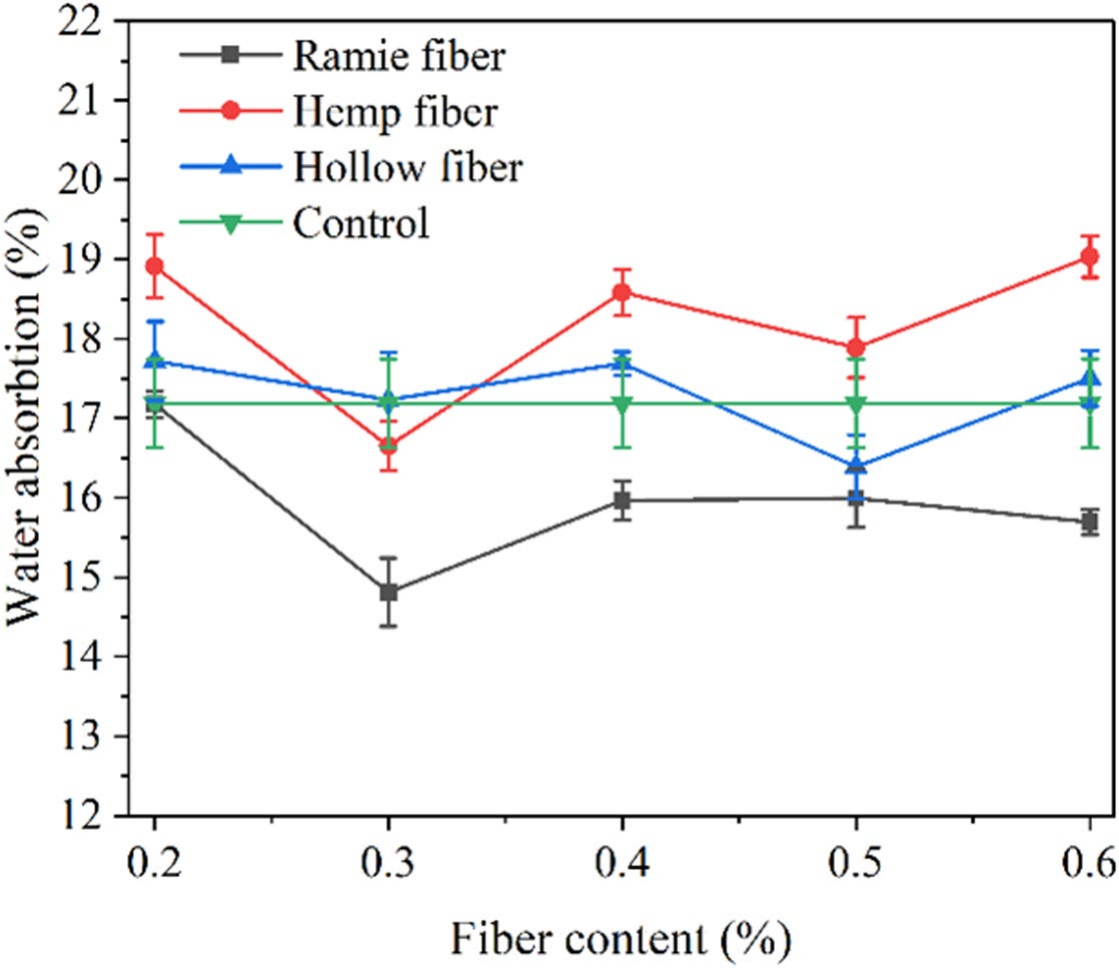
Water absorption of the specimens.

The water absorption characteristics of the fiber-reinforced biocemented sand specimens are presented in Figure 4. For all fiber types, water absorption decreased with increasing fiber content. This trend continued until a specific content level was reached, beyond which water absorption increased, indicating the existence of an optimal fiber content that maximizes the water resistance of the biocemented calcareous sand. The minimum water absorption for the ramie fiber biocemented sand (RF), hemp fiber biocemented sand (HF), and hollow fiber biocemented sand (HoF) was 0.3, 0.3, and 0.5%, respectively, corresponding to reductions of 13.84, 3.14, and 4.65% in water absorption relative to the MICP-treated control samples. Among the three fiber types, ramie fiber-reinforced samples exhibited the greatest reduction in water absorption, likely due to the combined effects of pore filling by precipitated CaCO₃ and the physical obstruction of water pathways by the dispersed fibers. Hemp fiber was the least effective, as its high hydrophilicity often led to greater water retention than in the control. Hollow fiber achieved the lowest absolute absorption, with its unique structure creating a more tortuous path for water, though it required a higher content for optimal performance. Similar reductions in moisture absorption have been reported in other MICP fiber studies, where the addition of fiber facilitated interparticle bridging and reduced pore connectivity (Zhao et al., 2021).

### 3.2 Calcium carbonate results

The calcium carbonate precipitation rate was measured to evaluate the biocementation efficiency of each fiber type. The precipitation increases with increasing fiber content, then decreases after reaching an optimal point. At low contents, the limited fiber surface area limits the availability of nucleation sites for calcite crystallization. At high contents, fiber agglomeration hindered nutrient distribution and suppressed carbonate precipitation. Hollow fiber achieved the highest precipitation, reaching 20.85% at 0.5% content, representing a 19.5% improvement over the control (17.45%). Both ramie- and hemp-reinforced samples peaked at 0.3% content, achieving 19.65 and 19.01%, respectively. Ramie-reinforced specimens maintained relatively stable yields (18.55–19.65%) up to 0.6%, while hemp-reinforced specimens dropped to 17.10% at the same dosage. These results are consistent with those of van Paassen et al. (2010), who emphasized the role of nucleation sites and spatial distribution in CaCO₃ precipitation. The correlation between CaCO₃ content and mechanical properties is further analyzed in Section 3.6.

### 3.3 Bacterial retention rate results

**Figure 5:**
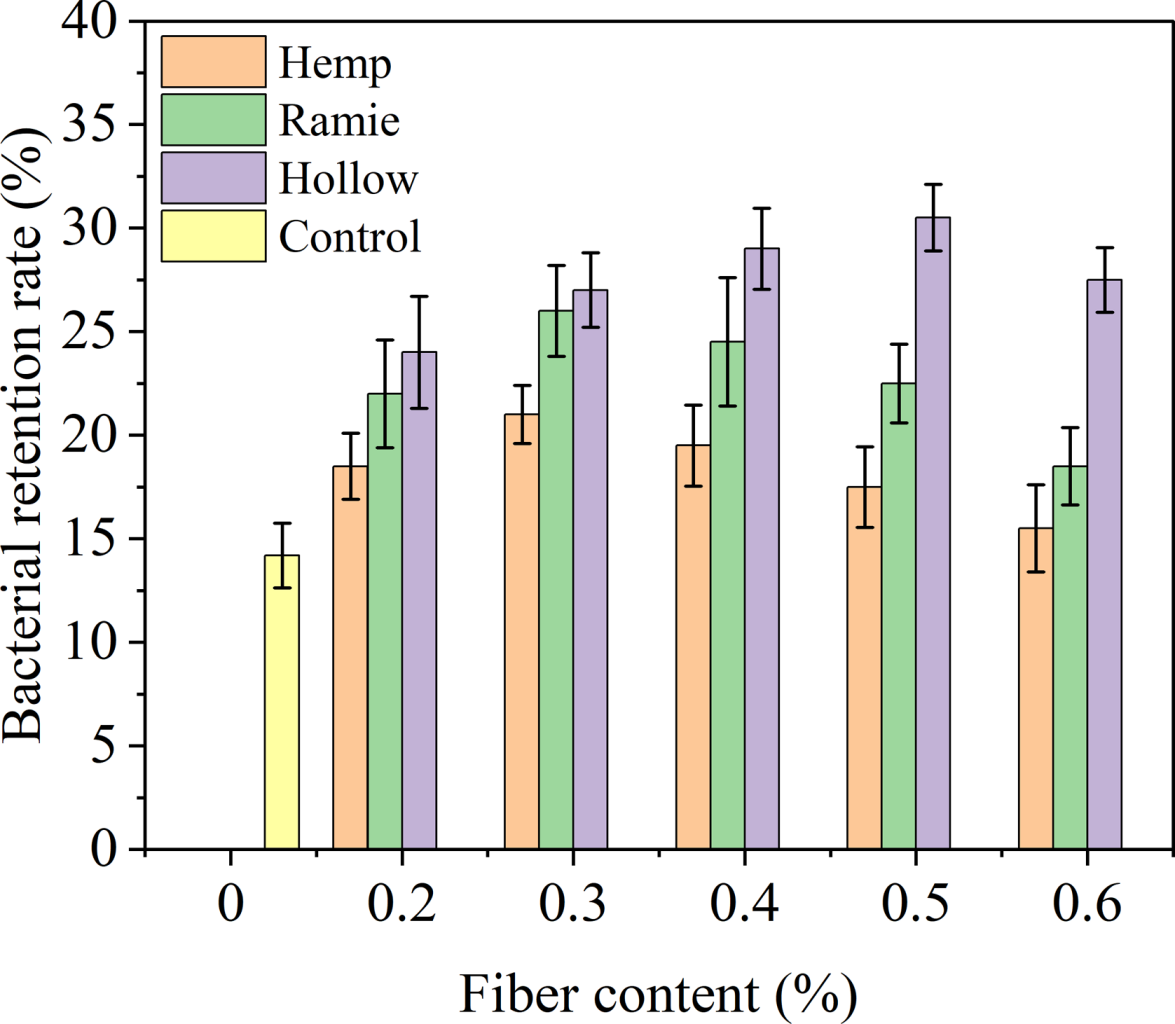
Bacterial retention rates of MICP-treated calcareous sand reinforced with hemp, ramie, and hollow fibers at varying fiber contents.

Figure 5 shows the bacterial retention rates for all fiber types and contents. The control sample exhibited a retention rate of 14.2%. For all three fibers, bacterial retention first increased with increasing fiber content, reached a peak, and then decreased. This trend can be explained by two competing mechanisms: at low levels, insufficient fiber surface area limits bacterial attachment; at high levels, fiber agglomeration reduces the effective surface area and limits bacterial mobility. Hollow fiber had the highest retention rate, reaching 30.5% at 0.5% content, an increase of 114.8% over the control. Ramie fiber reaches a peak retention rate of 26.0% at 0.3% content (an increase of 83.1%), while hemp fiber reaches a maximum retention rate of 21.0% (an increase of 47.9%) at a content of 0.3%. The excellent retention capacity of hollow fibers is attributed to their porous microstructure, which provides abundant attachment sites for bacterial adhesion through physical entrapment within the fiber lumen and surface irregularities (Yang et al., 2026). The rough, fibrillated surface of ramie also promotes bacterial adhesion, while hemp showed the lowest retention rate among the three fibers. The decrease beyond the optimal content is attributed to fiber agglomeration, which reduces the effective surface area for bacterial attachment and limits bacterial mobility.

### 3.4 Stress–strain curve of UCS test results

**Figure 6:**
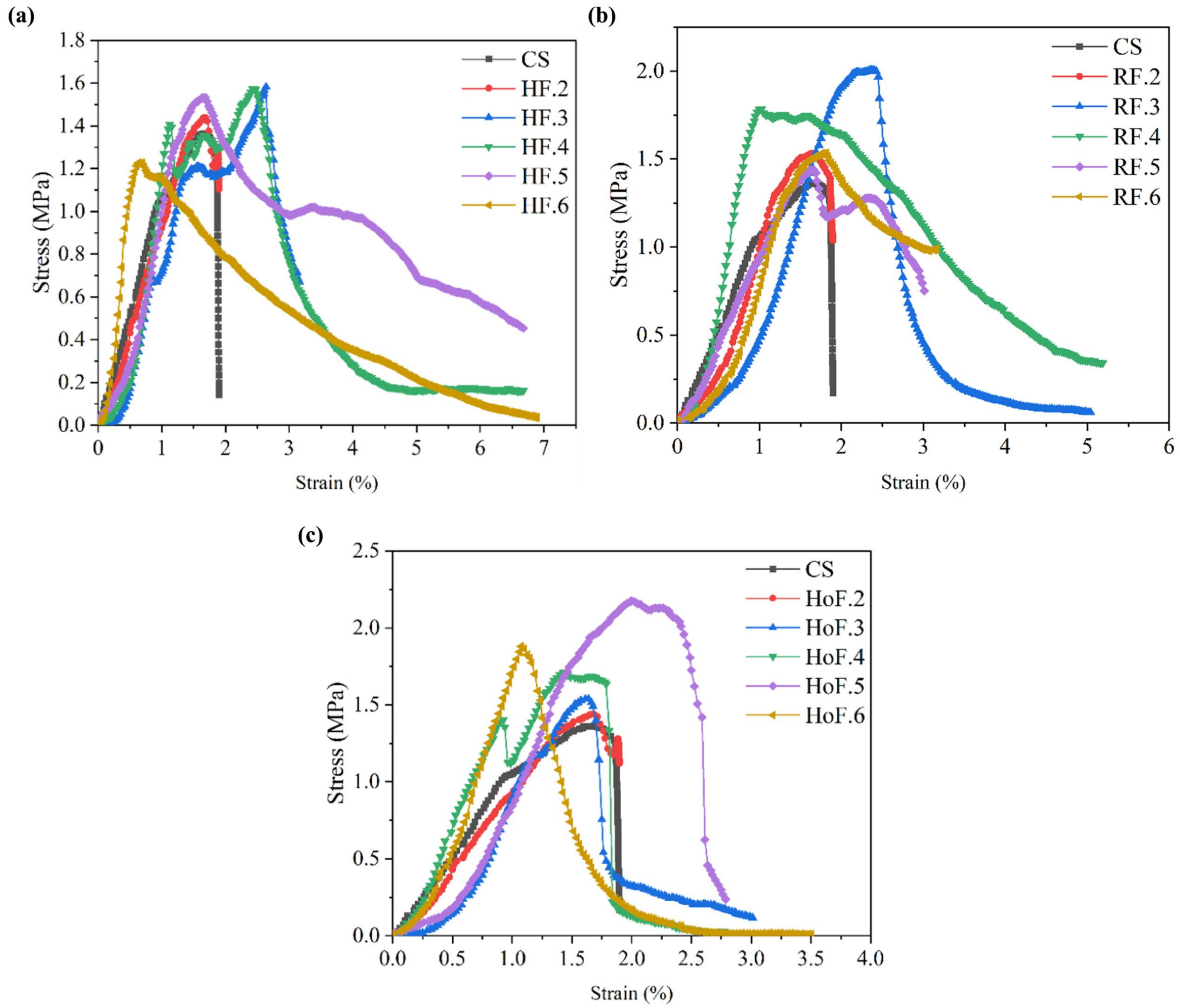
UCS stress–strain curves of biocemented sand under different fiber types and contents. **(a)** Hemp fibers, **(b)** ramie fibers, **(c)** hollow fibers.

The stress–strain curves of the different fiber-reinforced biocemented sand samples obtained from unconfined compression tests are shown in Figure 6. All the samples exhibited an initial elastic stage, during which the stress increased linearly with strain until reaching peak strength. After reaching this peak, the samples started to crack and sustain damage. It is worth noting that after the stress of the biocemented control samples (without fiber) reaches its peak, it drops sharply to zero, exhibiting a typical brittle failure mode. Previous studies have shown that soil samples with low fiber content exhibit two-stage stress–strain behavior, characterized by a rapid rise in stress followed by a sudden drop, implying brittle failure. On the other hand, samples with higher fiber content exhibit a third residual stress stage, in which fibers bridge cracks and sustain part of the load after failure (Shao et al., 2014; Chaosheng et al., 2007). This behavior was also observed in the present study, particularly in samples HF.4 and HF.5 in Figure 6B, where the reinforced calcareous sand sustained greater deformation while maintaining load-bearing capacity. The improvement became more pronounced with increasing fiber content, effectively delaying the overall failure of the samples. This behavior is primarily due to fibers acting as a “bridge” across cracks, capable of bearing specific tensile stress and preventing further crack progression. This indicates that the fibers were highly effective at immediately bridging cracks upon the sample’s initial failure, providing a significant and instantaneous improvement in ductility.

### 3.5 Unconfined compressive strength test results

The experimental results show that MICP treatment effectively solidifies calcareous sand. As a result, the unconfined compressive strength of the control sample was approximately 1.36 MPa. The UCS was found to be highly dependent on the fiber content. When the fiber content exceeded a specific value, the UCS decreased. For example, the UCS of hemp samples increased from 1.44 MPa at a 0.2% fiber content to 1.62 MPa at a 0.3% fiber content, representing an increase of 12.83% compared to the control sample. However, after exceeding 0.3%, the UCS decreased to 1.57 MPa at 0.4% and further reduced to 1.23 MPa at 0.6%. The optimal UCS of HF was achieved at a fiber content of 0.3%, at which the greatest strength improvement was observed. Similar results were obtained for ramie fiber and hollow fiber. In summary, UCS values follow a similar trend across fiber types, with the highest UCS observed at 0.3% fiber content for both HF and RF, and at 0.5% fiber content for HoF. The difference in optimal fiber content between natural fibers (0.3%) and hollow fibers (0.5%) can be explained by different material properties. Natural fibers have rougher surfaces and higher inter-fiber friction. On the other hand, hollow fibers are smoother and more rigid, reducing tangling and enabling higher dosages. Furthermore, the lower density of natural fibers results in a higher fiber volume occupation and increases the risk of weak areas. Finally, the porous structure of hollow fibers enhances bacterial retention and CaCO₃ nucleation, compensating for the downsides of higher fiber content. Among all fiber types, HoF showed the highest UCS increase of 59.54% at its respective optimal fiber content, followed by RF with a 46.83% increase and HF with a 12.82% increase. This trend in strength is closely linked to the amount of calcium carbonate precipitated, as detailed in the following section.

### 3.6 UCS versus calcium carbonate

**Figure 7:**
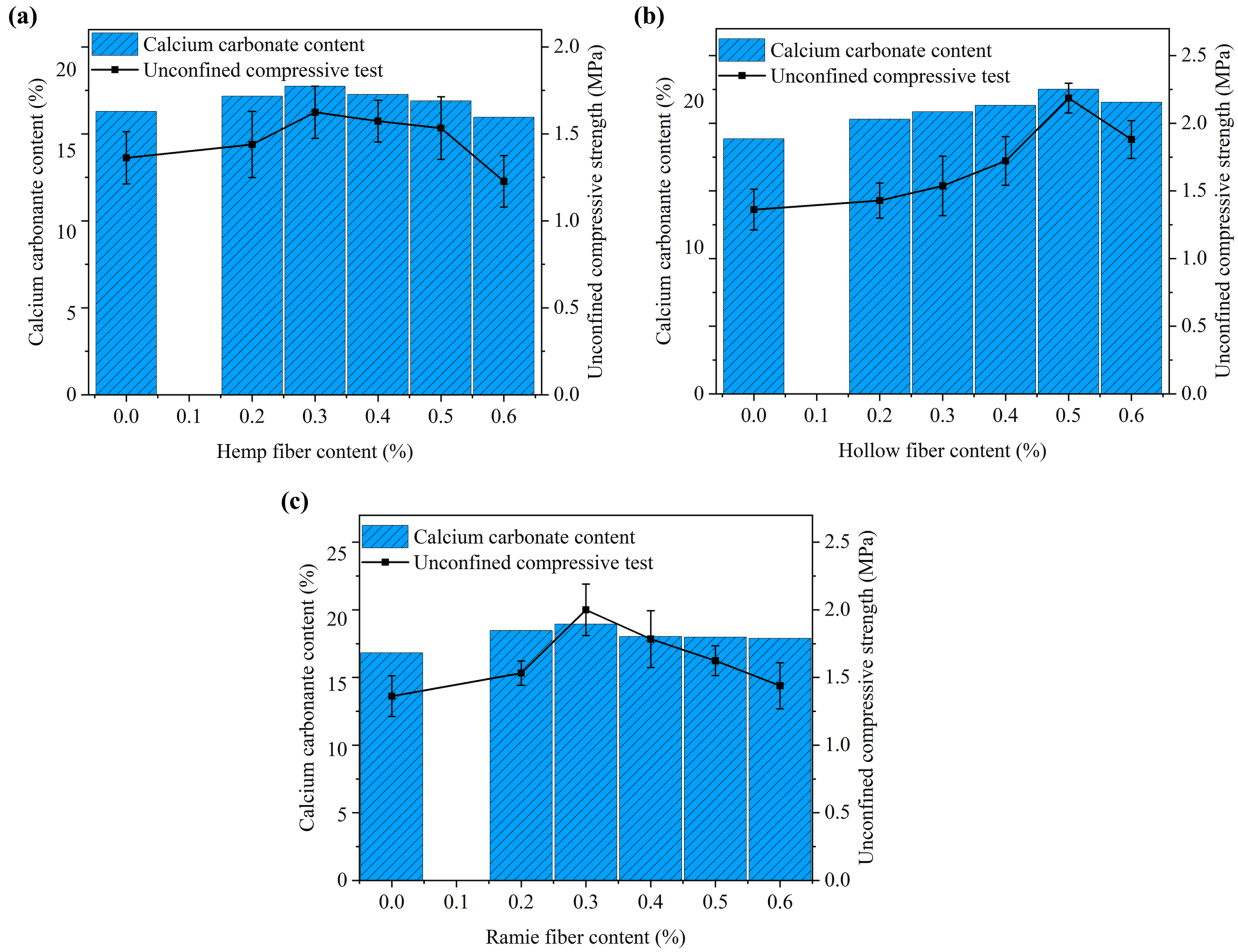
The dependence of calcium carbonate content and unconfined compressive strength of biocemented sand samples on varying fiber contents. **(a)** Hemp fiber, **(b)** ramie fiber, **(c)** hollow fiber.

The amount of calcium carbonate precipitated plays a crucial role in determining soil behavior. Figure 7 shows the relationship between calcium carbonate content and soil unconfined compressive strength (UCS) as a function of fiber content. For ramie fiber-reinforced samples with 0.3% fiber content, the calcium carbonate content reaches 19.65%, and the UCS is 2.0 MPa. This compares to the control sample (0% fiber), which had values of 17.45% and 1.36 MPa, respectively. The same tendency has been found for hemp fiber-reinforced samples. At 0.3% fiber content, the calcium carbonate content reaches its maximum value of 19.01%, and the UCS reaches 1.62 MPa. At a 0.6% fiber content, the calcium carbonate content decreases slightly to 17.62%, and the UCS drops to 1.23 MPa. Among all the fibers, the hollow fiber-reinforced samples exhibited the greatest improvement. The maximum values of 20.85% and 2.18 MPa were achieved for calcium carbonate content and UCS, respectively, at a fiber content of 0.5%. The observed correlation between calcium carbonate content and UCS is consistent with previous studies, which demonstrate that both the quantity and spatial distribution of precipitated CaCO_3_ are key factors governing the mechanical response of treated soils (Al Qabany et al., 2012; Cheng et al., 2013; Choi et al., 2016; Xiao et al., 2019).

Similarly, Abbasi (2025) reported a positive correlation between calcite content and mechanical properties in MICP-treated silica sand, with the best performance at 14.98% calcite content, corresponding to a UCS of 1,030 kPa and an E50 of 389 MPa. Furthermore, both calcite content and UCS increased with increasing number of treatment cycles, indicating that more CaCO₃ precipitation directly contributes to soil strength. These results indicate that CaCO₃ precipitation is the main driver of strength development in MICP-treated sand. A comparison of bacterial retention, calcium carbonate content, and unconfined compressive strength (UCS) demonstrates a consistent relationship among these parameters. For each fiber type, the fiber content that resulted in the greatest bacterial entrapment also produced the highest CaCO₃ precipitation and compressive strength. This pattern was most pronounced for hollow fibers, which exhibited the highest values across all three parameters. Yang et al. (2026) also reported that the hollow fiber membrane increased the calcium carbonate generation rate by 37.7% and UCS by 48.13%. These findings indicate that bacterial retention is a key factor affecting the efficiency of microbially induced calcium carbonate precipitation (MICP) treatment. Increased bacterial retention enhances calcite precipitation and supports the formation of a more effective fiber–calcite–sand cementation network. Therefore, the observed strength improvements can be attributed to enhanced bacterial retention, leading to increased CaCO₃ precipitation, as well as to improved biocementation efficiency promoted by the reinforcing fibers.

### 3.7 Failure analysis of biocemented sand

**Figure 8:**
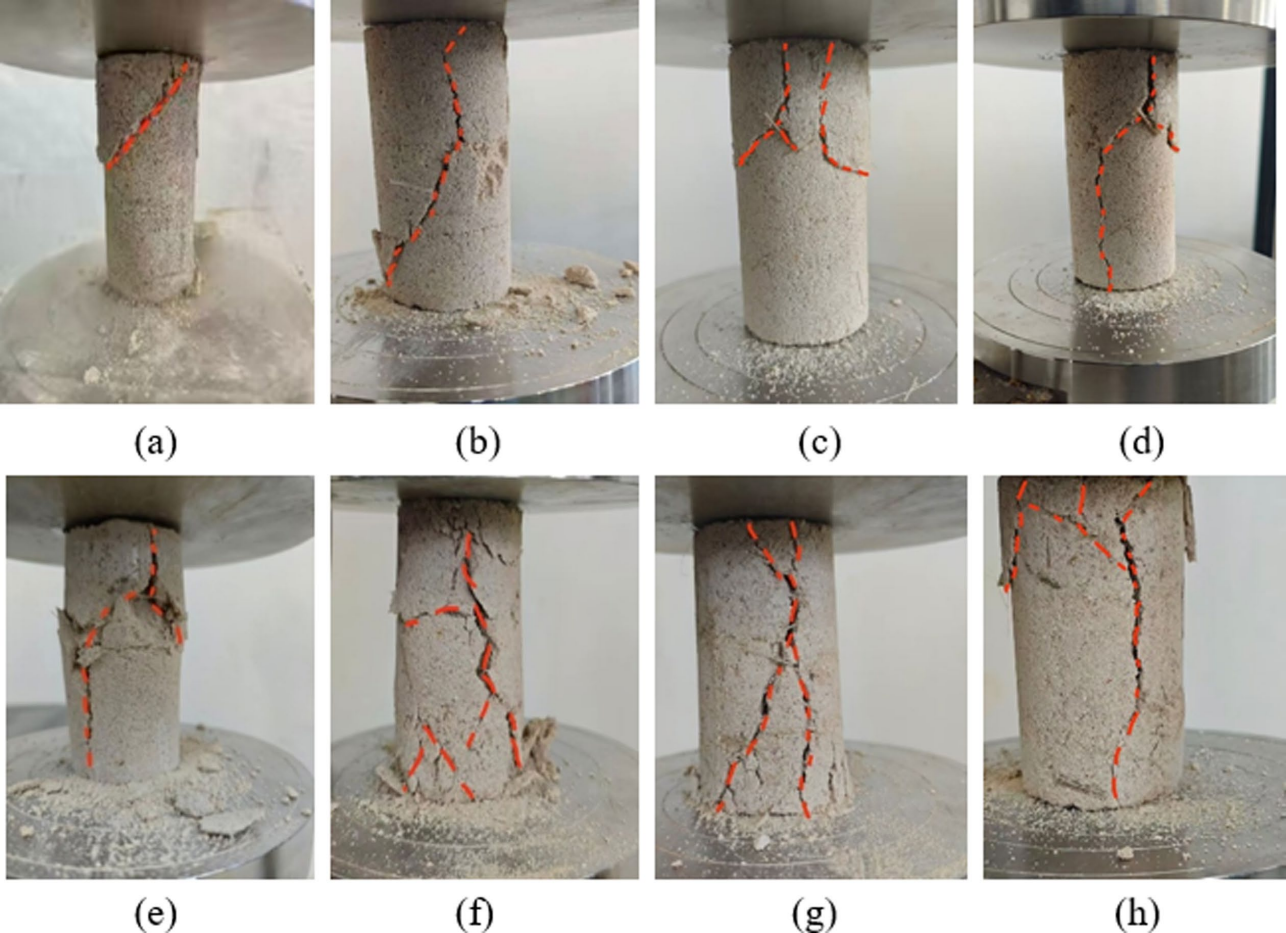
Unconfined compression failure modes of biocemented sand with **(a)** 0% fiber content, **(b)** 0.2% hollow fiber content, **(c)** 0.2% ramie fiber content, **(d)** 0.2% hemp fiber content, **(e)** 0.4% ramie fiber content, **(f)** 0.5% hemp fiber content, **(g)** 0.6% ramie fiber content, **(h)** 0.6% hemp fiber content.

Figure 8 illustrates the failure mechanism of biocemented sand. The failure behavior of MICP-treated calcareous sand is influenced by both the degree of cementation and the fibers’ reinforcing mechanism. Untreated samples exhibit classic brittle failure behavior, characterized by sudden cracking and rapid stress release. After MICP treatment, the samples exhibited a more cohesive, blocky failure pattern, resulting from calcium carbonate bonds formed between sand grains, thereby increasing the overall integrity of the specimen. The inclusion of fibers visibly altered the failure characteristics of the biocemented specimens. As the fiber content increases, the failure mode shifts from a sudden brittle fracture to a more progressive deformation pattern. This transition can be explained by fibers connecting cracks and spreading stress into the specimen area, allowing the specimen to withstand higher strains before failure. At lower fiber contents, fibers appeared well dispersed and effectively anchored sand particles within the cemented matrix, thereby improving deformation resistance. When fiber content exceeds the optimal value, a tendency for fiber clustering and uneven distribution occurs. This results in local stress concentrations at weak interfaces that promote premature failure. These localized weak zones reduce the overall load-bearing capacity and can produce mixed failure modes combining tensile splitting and shear deformation. The observed transition in failure behavior is consistent with the results of Xu et al. (2021), which show improved post-peak behavior. Furthermore, Liang et al. (2022) reported that strength gains reverse at high fiber contents due to fiber agglomeration and uneven CaCO₃ distribution. At high fiber concentrations, irregular distribution and aggregation lead to weak interfaces, illustrating the fiber agglomeration effect proposed by Park et al. (2014). The analysis reveals that the influence of fiber-matrix interactions on post-peak behavior is well controlled, and the fiber dispersion is optimal for imparting strength and ductility to the biocemented calcareous sand, enabling its proper utilization.

### 3.8 SEM analysis

**Figure 9:**
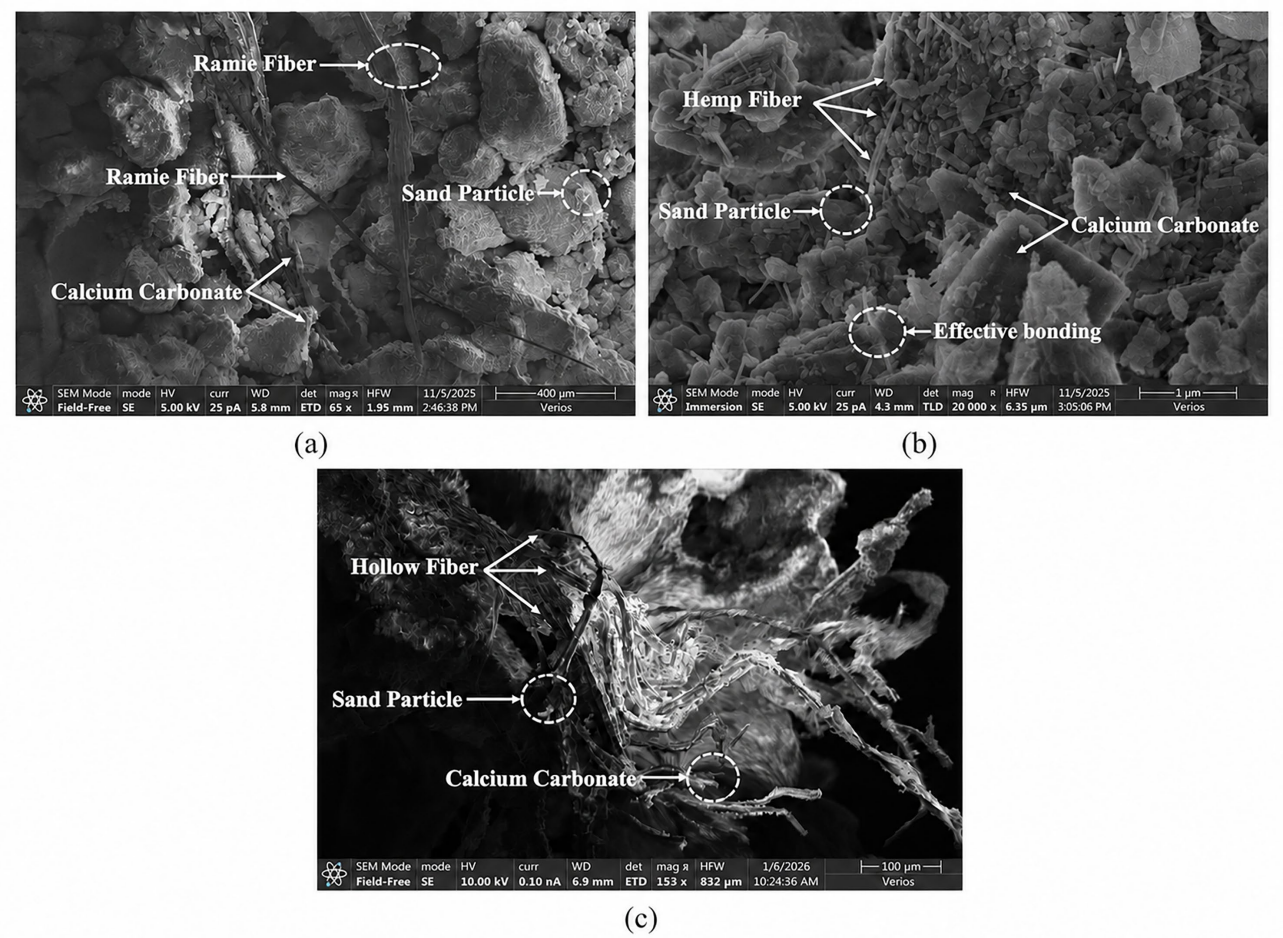
SEM images of fiber biocemented sand. **(a)** 0.2% ramie fiber content, **(b)** 0.3% hemp fiber content, **(c)** 0.4% hollow fiber content.

The microstructural characteristics of sand treated with MICP using hemp, ramie, and hollow fibers were studied using SEM analysis and are presented in Figure 9. The SEM images indicate that the effectiveness of bonding between the fibers and the bio-cemented sand structure primarily determines the UCS. The primary mechanism is the precipitation of calcite at the fiber-sand interface, which creates strong crystalline bridges that enhance stress transfer and matrix cohesion. For hollow fibers, SEM micrographs show that the surface of the hollow fiber membrane is extensively covered by calcium carbonate crystals, forming a dense crystalline layer. The hollow structure of the fibers provides favorable attachment sites for bacteria, promoting bacterial colonization and subsequent MICP reactions on the membrane surface. Bacterial retention measurements confirmed this mechanism, under optimal conditions (0.5% content), hollow fibers achieved a retention rate of 30.5%, representing a 114.8% increase over the control (14.2%). This robust anchoring ensures that the fiber’s full tensile capacity is mobilized, leading to a high UCS failure and a failure mode characterized by sand crushing and potential fiber rupture.

Similarly, ramie fibers exhibit extensive calcite precipitation on their naturally rough and fibrillated surfaces, creating a continuous, strong bond with the sand grains that results in comparable high UCS performance. On the other hand, the unconfined compressive strength of biocemented sand with hemp fibers is relatively low compared to that of other fiber types. This behavior may be attributed to the relatively weak interfacial bonding between the hemp fibers and the surrounding sand grains, resulting in poor stress transfer within the reinforcing matrix.

### 3.9 Engineering implications and practical applicability

The results of this study indicate that fiber-induced microbial calcium carbonate precipitation (MICP) treatment significantly improves the mechanical properties of calcareous sand. This improvement is achieved through increased bacterial retention, increased calcium carbonate precipitation, decreased water absorption, and increased compressive strength. These strength improvements suggest that the treatment is suitable for moderate soil stabilization applications such as improving coastal foundations, reinforcing embankments, preventing erosion, and improving soil for soft infrastructure projects. The three types of fiber studied have several practical advantages. Hemp and ramie fibers are renewable, biodegradable, and widely available natural materials, making them attractive options for sustainable geotechnical applications. Their relatively low cost and reduced environmental impact are consistent with the goals of green soil improvement technologies. However, natural fibers may be susceptible to long-term biodegradation under certain environmental conditions, potentially limiting their durability in harsh field environments.

Hollow fibers demonstrated the most pronounced performance in terms of bacterial retention, calcium carbonate precipitation, and increased strength compared to the other fibers examined. Its porous architecture facilitates bacterial adhesion and calcite formation, thus allowing more effective biocementation. Although the production of synthetic hollow fibers may incur higher initial material costs, their greater effectiveness could decrease the number of treatment cycles required to achieve desired engineering properties, which could ultimately increase cost-effectiveness. Water absorption test results indicate that incorporating fibers into the MICP treatment helps minimize pore connectivity and limit moisture penetration, potentially improving long-term durability. However, it is important to note that this study was conducted in a controlled laboratory environment. To pave the way for large-scale application, further research is needed to evaluate factors such as long-term durability, effects of environmental exposure, consistency of field-scale processing, and economic feasibility under realistic construction scenarios.

## 4 Conclusion

This study investigated the reinforcement of calcareous sand from South China Sea islands and reefs using MICP technology combined with hemp, ramie, and hollow fibers. A series of fiber-reinforced, MICP-treated calcareous sand samples with varying fiber types and contents (0.2, 0.3, 0.4, 0.5, and 0.6%) were prepared and subjected to water absorption tests, bacterial retention measurement, unconfined compression tests, and analysis of calcium carbonate content. Additionally, scanning electron microscopy (SEM) analysis provided microscopic insight into the interactions between the fibers and calcium carbonate precipitates on the sand particles. The experimental results and microscopic observations of different samples lead to the following major conclusions.

(1) The combined use of fiber reinforcement and MICP enhanced the efficiency of the biocementation process by improving bacterial retention and promoting calcium carbonate precipitation. For all fiber types, bacterial retention and CaCO₃ content initially increased and then decreased with increasing fiber content, suggesting an optimal dosage. Hollow fibers (0.5% content) achieved the highest bacterial retention of 30.5% (a 114.8% increase over the control) and a CaCO₃ content of 20.85% (a 19.5% increase), with water absorption reduced by 4.65%. The Ramie fibers (0.3% content) reached a retention of 26.0% (an 83.1% increase), a CaCO₃ of 19.65% (a 12.6% increase), and the most substantial water absorption reduction of 13.84%. Hemp fibers (0.3% content) showed retention of 21.0% (a 47.9% increase), CaCO₃ of 19.01% (an 8.9% increase), and water absorption reduced by 3.14%.
(2) Water absorption tests showed a decrease in water absorption for all fiber-reinforced samples, confirming the improvement in pore densification. At their respective optimal dosages, ramie fiber (0.3%) showed the largest decrease (13.84%), followed by hollow fiber (0.5%) (4.65%) and hemp fiber (0.3%) (3.14%). The large reduction in ramie-reinforced samples was attributed to the effective pore filling of precipitated CaCO₃ and physical blocking of water channels by dispersed fibers, which was promoted by the rough fibrillated surface morphology of ramie. Hemp fiber showed the smallest reduction, possibly due to its high hydrophilicity, which offsets the pore-clogging effect. Reduced water absorption indicates enhanced durability-related properties of fiber-reinforced samples—a key consideration for fiber-reinforced samples.
(3) The unconfined compressive strength (UCS) of the treated samples increases with increasing fiber addition, reaches an optimal level, and then decreases at higher fiber content due to the fiber agglomeration effect. The UCS value of the hollow fiber-reinforced sample with 0.5% fiber content was the highest at 2.18 MPa, an increase of 59.54% compared to the control sample (1.36 MPa). Ramie fiber reaches 2.00 MPa at 0.3% content (46.83% increase), while hemp fiber reaches 1.62 MPa (19.12% increase) at 0.3% content. The similarity between UCS and CaCO₃ trends confirms the key role of calcium carbonate precipitation in controlling strength development.
(4) Scanning electron microscopy analysis shows that hollow fibers and ramie fibers promote the extensive precipitation of calcite and form a strong interface combination with sand particles to form a more stable cement-soil skeleton. In contrast, hemp fibers exhibit relatively weak interfacial bonding, which corresponds to the lowest strength improvement among the fibers studied. The strength enhancement mechanism is mainly attributed to the formation of the composite sand-calcium carbonate-fiber network, in which calcite precipitation, fiber bridging, and interfacial bonding act synergistically to improve the mechanical properties of the treated sand.

The addition of fibers enhances the reinforcement effect and improves the uniformity of MICP treatment. However, this technology requires further development to move from laboratory testing to practical applications. The superior properties of ramie fiber necessitate specialized research to develop surface treatments, such as alkali and silane treatments, to enhance its bond strength and environmental resistance in seawater. Optimization of construction methods, including injection techniques, nutrient levels, and fiber placement, as well as comprehensive mechanical testing, such as shear strength and split tensile strength, is also required to maximize performance and economics for commercial use.

## Data Availability

The original contributions presented in the study are included in the article/supplementary material, further inquiries can be directed to the corresponding author.
